# Medical and Philosophical Intelligence

**Published:** 1817-03

**Authors:** 


### MEDICAL AND PHILOSOPHICAL INTELLIGENCE.

*To the Editors of the London Medical and Physical Journal.*

GENTLEMEN,

YOUR correspondent, E. I. C., was pleased to make a reply to my "Remarks on the Use of the Minim Measure;" and, if *he* prefers an anonymous letter to one with his signature affixed, or, in other words, if he likes a false name, I must request he will not again be guilty of putting a wrong construction on my future communications, which, in the present case, he will see he has done. By what I said in my letter, I do not wish it to be understood—I consider the practice of dropping fluid from bottles a good one; on the contrary, I always thought it incorrect, (as one must naturally suppose, the difference in the size of the neck of a bottle must influence the dropping the fluid.) The ground on which I attach blame to the College of Physicians, is not for introducing the minim measure, but for not making it so public as was certainly necessary to guard the practitioners from error. That the minim is the sixtieth part of a fluid-drachm, I did not stand in need of the information conveyed by E. I. C.'s letter to acquaint me. There are yet many practitioners, who consider the minim and the drop as synonymous, and order and dispense prescriptions accordingly. As E. I. C. might have seen, from Mr. Walker's letter in the last Number of the Medical Journal.

I am sorry to make the pages of your work a vehicle for controversy, which can be no interest to your numerous readers; but, the wish to justify myself in this point, has induced me to trouble you with this reply.

I have the honor to be, gentlemen,

Yours, &c. &c.

*Sloane-street, Jan. 3,* 1817. HENRY WARD.

*Report*

*Report of the City of London Truss Society, for the Relief of the Ruptured Poor, throughout the Kingdom, Grocers'-hall Court, Poultry.*—From the most accurate estimation, it appears, that this malady exists in one person in eight through the whole male population of this kingdom, and even in a much greater proportion among the labouring classes of the community, in manufacturing districts, particularly in those persons who are employed in weaving, or on the water as boatmen.

In one district in the western part of England, comprising 200,000 inhabitants, it has been ascertained, by actual observation, that upwards of one in five of the whole population labour under this malady.

| | | |
|---|---|---|
| Patients relieved by this Society in | 1808 | 227 |
| | 1809 | 570 |
| | 1810 | 813 |
| | 1811 | 1094 |
| | 1812 | 1666 |
| | 1813 | 1800 |
| | 1814 | 2064 |
| | 1815 | 2473 |
| | 1816 | 2610 |
| | | 13,317 |

The following statement of the situation and occurrence of hernia, at different periods of life, has been extracted from the register of the patients relieved by the City of London Truss Society, within the short period of nine years.

In 12,282 cases, 10,121 were males, and 2161 were females.

| Males. | Females. | | | |
|---|---|---|---|---|
| 2050 | 40 | left inguinal | 5761 inguinal | 6621 single. |
| 3629 | 42 | right inguinal | | |
| 68 | 359 | left femoral | 860 femoral | |
| 76 | 357 | right femoral | | |
| 3951 | 31 | double inguinal | | 4286 double |
| 61 | 243 | double femoral | | |
| 45 | 472 | umbilical | | 603 |
| 24 | 62 | ventral | | |
| | 1 | perineal | | 1 |
| | 2 | obturator | | 2 |
| 18 | 30 | have undergone operations, all except *one* have been completely successful | | 48 |
| 110 | 111 | with umbilical and inguinal hernia have been cured | | 221 |
| 84 | 19 | with prolapsus ani | | 103 |
| | 390 | with prolapsus uteri | | 390 |
| 5 | 2 | with varix of the abdominal veins | | 7 |
| 10121 | 2161 | —12282 | | 12282 |

| | | | | | | |
|---|---|---|---|---|---|---|
| 875 | patients were relieved with trusses under 10 years of age. | | | | | |
| 609 | ditto, | between | 10 | and | 20 | ditto. |
| 1219 | ditto, | ——— | 20 | and | 30 | ditto. |
| 2032 | ditto, | ——— | 30 | and | 40 | ditto. |
| 2165 | ditto, | ——— | 40 | and | 50 | ditto. |
| 2160 | ditto, | ——— | 50 | and | 60 | ditto. |
| 1522 | ditto, | ——— | 60 | and | 70 | ditto. |
| 509 | ditto, | ——— | 70 | and | 80 | ditto. |
| 67 | ditto, | ——— | 80 | and | 90 | ditto. |
| 4 | ditto, | ——— | 90 | and | 100 | ditto. |
| 11162 | | | | | | |

Six females each had umbilical and double femoral hernia, 2 females had large ventral and double femoral hernia, 5 females had umbilical and ventral hernia, 1 female had ventral and prolapsus uteri, 6 females had umbilical and single femoral hernia, 4 females had right inguinal congenital hernia, 7 females had left inguinal and right femoral hernia, 1 female had left femoral and right obturator hernia, 4 females had ventral and single femoral hernia, 1 female had double femoral and right inguinal hernia, 6 females had double inguinal and umbilical hernia, 1 female had right inguinal and ventral hernia, 7 females had left inguinal and left femoral hernia.

Twenty-six males each had left femoral and right inguinal hernia, 8 males had left inguinal and left femoral hernia, 21 males had left inguinal and right femoral hernia, 9 males had double inguinal and right femoral hernia, 6 males had double inguinal and left femoral hernia, 4 males had right inguinal and right femoral hernia, 2 males each had double inguinal and double femoral hernia, 6 males had double femoral and right inguinal hernia, 3 males had ventral and right inguinal hernia, 1 male had ventral and right femoral hernia, 2 males had ventral and left inguinal hernia, 1 male had ventral and double inguinal hernia, 1 male had left inguinal, umbilical, and ventral hernia, 1 male had umbilical and left inguinal hernia, 1 male had umbilical and left femoral hernia, 1 male had umbilical and right femoral hernia, 8 males each had umbilical and double inguinal hernia, 2 males had double femoral and right inguinal hernia; 627 patients had congenital hernia; 5 males had very large varicose veins on the abdomen.

---

Mr. Curtis has introduced Artificial Ears for Deaf Persons from France, where they were manufactured. By being closely adapted to the ear, they increase the collection of sound; but, besides the collection of sound, there is an additional force wanted to transmit it through the passage. In this respect the French invention is deficient. To remedy this defect, a small tube is added, which, by contracting the passage, will occasion the sound to enter with greater force. The form of this invention is particularly convenient, being applied over the natural ears, which they are

4 made made to resemble. The same gentleman has also invented a hearing trumpet, forming a parabolic conoid, on the same principle as the speaking trumpet used at sea, which is so well known to answer the purpose in extending the impression of sound. It has this convenience, that it shuts up in a small case for the pocket.

---

*Annual Report of the Hospital for the Small-pox, for Inoculation, and for Vaccination, at Pancras.*

An Account of the Number of Patients admitted into the Hospital, and the Number of Out-Patients Vaccinated; also the Number of Mothers admitted as boarders to Nurse their Children, from January 1, 1816, to January 1, 1817.

| 1816. | Patients admitted with the Natural Small-pox. | for Inoculation. | for Vaccination. | Out-Patients for Vaccination. | Mothers admitted to Nurse their Children. |
|---|---|---|---|---|---|
| January | 7 | 2 | | 77 | |
| February | 5 | 4 | | 51 | |
| March | 5 | 3 | | 163 | |
| April | 5 | 1 | | 321 | |
| May | 17 | 12 | | 369 | |
| June | 17 | 2 | | 298 | |
| July | 14 | 8 | | 224 | |
| August | 9 | 2 | | 252 | |
| September | 8 | | 3 | 244 | |
| October | 12 | 2 | | 175 | |
| November | 15 | 1 | | 80 | |
| December | 27 | | 2 | 59 | |
| | 141 | 37 | 5 | 2313 | 11 |
| Died | 27 | | | | |

JOHN CHRISTIAN WACHSEL,

*Apothecary and Steward.*

*Jan.* 2, 1817.

This makes the mortality about one in six, which, considering that the worst cases are sent to that house, may be considered a very small ratio.

The following case is interesting, as exhibiting the advantage of vaccinating after a long exposure to small-pox.

Charlotte Parry, aged 25 years, a married woman, residing at No. 8, Plumtree-court, Shoe-lane, with her infant, Sarah, aged six six months, at her breast, was sent to the Small-pox Hospital at Pancras with the following certificate:—"This is to certify that Charlotte Parry, wife of William Parry, has the confluent small-pox, and has an infant at her breast, and is a deserving object for the Institution. Signed Robert Davis, Surgeon, 126, Lower Holborn."

She was admitted January 25th, 1817, on the sixth day of the disease, and the third of the eruption. The child was perfectly well in health. Mr. Wachsel recommended it to the father to have the infant vaccinated: he hesitated a few minutes, as it had been exposed already to the infection of the natural small-pox. He also stated that his wife reported to him, that she had herself been inoculated for the cow-pox eight years ago. Such a report made Mr. Wachsel more desirous to vaccinate the child, that the prejudice against vaccination might be removed. The father consented to any means which might preserve his child from the small-pox, and it was immediately vaccinated with fresh vaccine fluid. The progress of vaccination on the tenth day was a complete areola. On the eleventh day the child was seen by Mr. Smith, a surgeon of Stoke Newington. On the twelfth day the arm began to dry; the areola continued; in the evening the child was restless, during the night feverish. On the thirteenth day a few elevated spots appeared on the face and right arm; some of them contained a little fluid. On the fourteenth and fifteenth days more vesicles appeared; the former began to dry. On the twenty-first the insertion in the arm and vesicles were all scaled; none of them contained any purulent matter. It was constantly in bed with the mother, who died on the 2d day of February. The infant was restored, in perfect health, by the Surgeon of the Hospital, to its father, on the 14th day of February.

P.S.—The left arm of the mother had a faint cicatrix, somewhat irregular.

---

*On the Influence of Moisture on the Irritability of the Muscles.*—Professor Nasse made some very interesting experiments on the effects of water on the irritable muscle, of which the following is the result. He cut off the thighs of several frogs, put some of them in water of 60° Fahrenheit, whilst he exposed some others to the influence of the atmosphere. The water not only changed the bulk and the weight of the muscle, but also made it whiter and more transparent, and gave it nearly the appearance of white wax, and, on its being pressed between the fingers, the water was not squeezed out again. The water diminished and destroyed its irritability, and much accelerated its destruction. The thigh exposed to the air, showed irritability from galvanism, after having been exposed to the air for ten or twelve hours, while that which had been put in water had left off contracting itself nearly two hours sooner. The water does not show this effect merely on account of its relation to caloric, or on account of its abstracting the blood, but, probably, through other means, produced by its penetration tion into the muscle. Many long known facts seem connected with this, as the muscular debility of dropsical limbs, the effect of tepid water-baths and of a moist atmosphere. Animals, which on account of the thinness of their skin, can offer but little resistance to the penetration of moisture, enjoy but little muscular strength, whereas birds and beast of prey possess a deal of it. The cause of these phenomena it is difficult to conjecture, as there are as yet so few established facts in regard to this, it is however not unlikely, that they may be connected with an electric relation of the living muscular fibre. The connection subsisting between muscular activity and electric tension is obvious, from the phenomena, and water may, perhaps, destroy that tension.

---

*The Blue Nose not always a Fatal Symptom in Typhus.*—Dr. Kraft (in Hufeland's Journal of 1815, page 109, &c. on Typhus Bellicus and the Blue Nose,) declares this symptom as a prognostic of its incurability, and even as a certain forerunner of death. This is contradicted by Dr. Von Wendelstadt. "The redness (says he) with which the blueness of the nose commences is unquestionably of the erysipelatous kind, confining itself in the commencement likewise to the nose, but spreading afterwards to the neck, breast, and navel, and then encompassing the body in that region, like the zona. Whether the nose, which certainly is an organ that particularly offers an area to the contagion, may be particularly affected, and suffer in particular by the general morbid state of the body, is as yet undecided. Whether the erysipelas does become phlegmonoides, and affects the brain, is also undecided. It is equally uncertain, whether this erysipelas is not, perhaps, a mere symptom of a latent greater disturbance in the process of animation, by perhaps interrupting the hepatic functions; neither has it been ascertained, whether it be not analogous to the sphacelous toes of old age. We know the blue nose as a phenomenon, and that is all we know about it."

Egenolff, jun. of Desax, a strong young peasant, brought with him a typhus from the hospital. The disorder was, all at once, very violent, and the nose became of a circumscribed bluish red. When the case was reported to me, the furious delirium and the bluish nose were marked in glaring colours as particular characteristics. According to the dark theory of the nexus of the plexus nervorum abdominis, particularly of the ganglion semilunaris, with the medulla cerebralis and dorsalis, I ordered a large blister to be applied to the whole belly, and to be kept on till it had fully drawn, and the patient recovered.

---

*The dangerous Effects of Extravasated Blood in the System.*—"I have long remarked, (says Baron Larrey) that, when the blood discharged out of its proper vessels, lodges in the cellular membranes, or any cavity of the body, it enters immediately, by the effect of repose and the absence of caloric and vitality, into a state of decomposition; the portion which coagulates disengages the the salts in acids contained in this fluid; they tend to new combinations, irritate the sensible parts, inflame them, and from whence all the effects of inflammation. The surgeon cannot, therefore, pay too much or too speedy attention to blood extravasated in the serous cavities, the shortest stay in which puts the life of the patient in danger. This remark shows how little the French surgeons have attended to Mr. Hunter's luminous remarks on the various circumstances under which blood is extravasated.

---

*Of Accouchement and the Ceremonies attending it among the Arabs.*—Throughout all Asia the business of an accoucheur is unknown, and, whatever art or address may be requisite, every thing is entrusted to a midwife, whose only skill is experience. These women, seated on the ground, take the woman in labour on their knees, and receive the burthen in a sieve placed between their thighs. No witness interrupts this operation, the presence of even the husband would be a stain on his honour. After the child is born, the midwife puts the mother to bed, and, calling the father of the infant before any one yet knows the sex, informs him of it through the veil that separates them, and receives from his hand a present which does not enter into her regular fees.

During this time, the relatives and friends, assembled either in a private tent or in the open air not far from the tent of the father, wait with impatience the news of the happy birth and the sex of the infant. No sooner has the husband been told by the midwife, than he hastens to them, and informs them of these two points. The men congratulate him, and the women give themselves up to a tumultuous joy, singing and piercing the air with the cry of *lililili!*

The father then presents the guests tobacco to smoke, coffee and water, and sugar when he has any, and afterwards a liquor made from fenugreek, honey, and dry dates. This beverage is also given to the mother as a cordial, which strengthens the viscera relaxed by the labour, and which, it is believed, has the virtue of provoking sanguine evacuations.

---

*On Wounds of the Cornea, attended by the issue of the Aqueous Membrane of the Iris, cured by a New Method*; by BARON LARREY. "This method was suggested to me by an accident which happened to my daughter, Charlotte Isaure. When the hernia of these membranes is the result of a wound made by a sharp instrument, it must be returned (very gently) immediately, by means of a gold style, with a knob or button at the point; any other metal applied to these delicate and very sensible parts might produce galvanic impressions very injurious to the patient. The membranes resume their regular position, and we thus prevent the deformity and troubled vision which ordinarily takes place when we abandon the staphyloma to the simple resources of nature.

Charlotte Isaure Larrey, ætat. about seven, cutting the bread at her breakfast, a piece of crust flew into her eye; she instantly raised raised her hand to her eye to remove it, and, unfortunately, plunged the point of the knife, newly sharpened, into the centre of the cornea, which was cut obliquely in half its external diameter. A portion of aqueous membrane, and even of the iris, presented themselves, and formed a hernia of the size of a pea; the aqueous humour had flowed, they sunk in, and the sight was totally suspended. I returned almost at the instant, and preserved sufficient courage and calmness to administer necessary aid. I returned, with my gold probe, or style, the membranous portions forming the staphyloma, and I endeavoured to replace them in their primitive state. After the entire reduction, I drew the eye-lid over the eye, and covered the eye then closed with proper compresses steeped in the vegeto-mineral water, and bandaged it in the usual manner. I ordered her feet to be bathed, gave refreshing beverages, confined her to a strict regimen, which, with keeping her in a dark room, sufficed to effect a perfect cure in a few days.

---

*Coppenhagen.*—At the last meeting of the Royal Medical Society, the following Treatises were read: On some of the Principal Disorders of the Nails and their Treatment, by Dr. Tenges. On a recently discovered Alimentary Substance called Iodin, by Mr. Jacobsen, head surgeon of the staff. On a Human Monster, where the Abdominal Integuments were wanting, and where most of the Organs deviated considerably from the normal Form; by Prof. Herholdt. On the Nature and Origin of the Inversio Vesicæ; Mr. Jacobsen.

The temporary president of the society is the Counsellor of State, Dr. Bang; vice-president, Prof. Mynster; secretary, Prof. Klingbergh. Prof. Reinhardt and Dr. Howitz have been created ordinary members; and accession to the assembly has been granted to Messrs. Somer, Holst, Lunding, Sleinbergh, and Dr. Raben, of Lund.

*Ibidem.*—The invitatory programme for the anniversary of the Institution for Deaf and Dumb, of the year 1815, held by Dr. and Prof. Castbergh, contained Remarks on the Disorders of the Deaf and Dumb; according to which, a scrofulous diathesis is predominant in a great many of them, and scrofula a common disease among these unfortunates. [We wish this paper may be followed by a definition of Scrofula.—English Edit.]

*Ibidem.*—According to the report of the committee of the Vaccine Establishment in Denmark, delivered to the chancery, for the years 1814 and 1815, being the fourteenth and fifteenth of its existence; the number vaccinated in these two years exceeded that of any of the preceding ones. In 1814, the number amounted to 23,392; of whom 17,040 were inoculated by medical men, 5,082 by clergymen and schoolmasters, and 1,270 by private persons. In 1815, the total was 24,425; of whom 20,011 were vaccinated by professional men, 3,699 by the clergy, and 715 by private persons. The Copenhagen Public Committee for Vaccination, sent abroad, in 1814, 208 portions of vaccine matter; and, in 1815, as as many as 246, of which about one-third went to Iceland, Greenland, the West India colonies, and the coast of Guinea. In Greenland and the Faröer Isles, vaccination is making very good progress, not so well does it go on in Iceland. On the proposal having been made, the professors of the Mosaic religion have also been subjected to the orders already passed.

---

*On the Duties and Acquirements necessary to Medical Students.*

Public Sitting of the Faculty of Medicine of Paris.—On Monday the 25th Nov. 1816, the Professors of the Faculty held an extraordinary sitting, in the presence of the royal Commissioners of Public Instruction, to distribute the prizes to the pupils of the Practical School.

Professor Dumeril, the President, delivered the customary oration, equally brilliant in literary composition, as it is important in its nature, enforcing the duties of medical pupils, and raising medicine to that elevated rank which it proudly claims amongst the natural sciences.

After tracing the history of the medical schools in France, and shewing that students flock from all parts, ' the sons of Albion and the sons of Iberia, youths from the Ganges and the banks of the Neva, or publicly deputed by the two Americas,' he adds, my imagination warms, and my French heart palpitates, on thinking that, when they return to their country, these pupils will carry with the knowledge they have acquired a fond remembrance of the masters who instructed them.

At this name of pupils, think, young disciples, on the duties imposed upon you by the Prince of medicine, think yourselves fortunate if you possess the qualities that he demands. He requires, for the art of healing, a great natural disposition, a good domestic education, taste, ardour for labour, youth, time, indefatigable patience, and a courage proof against every thing. All these qualities are precious, but, above all, cultivate the ardent desire to learn, which science itself will soon increase in you; and with this noble perseverance you will conquer every obstacle.

Let not him seek to inscribe himself in the number of the disciples of Hippocrates who does not feel the magnanimous sentiment of the divine old man who, when receiving with deference the deputies of a powerful monarch, sacrificed fortune to the love of his country, and rejected with dignity the marks of honour and the riches they laid at his feet.*

After having enumerated the qualities we wish to find in our pupils, I will give them some counsels, dictated in the name of my colleagues, to guide their uncertain steps in the career they wish to pursue; and, above all, to teach them how usefully to employ and economize their time, recollecting that *life is composed of it*,—

---

* Hippocrates refusing the presents of the King of Persia. An exquisite picture on this subject has recently been presented to the School of Medicine, painted by M. Girodet Trieson.

 the the wise maxim of a great philosopher, which we cannot too often impress upon the youthful mind.

Medicine is the science which teaches to remedy the various derangements that life may experience in its duration. The first study ought to be that of the series of phenomena which take place in an animated being when he resists those constant forces of Nature which alone rule and determine the existence of inert bodies. It is in the book of death that we learn to read the history of life. It is in decomposing her instruments that we discover at least some of the wheels which put them in motion. It is, therefore, necessary to study the structure of the body, before we explain its action.

At first, raise the veil which Nature has thrown upon our organization, study successively all the pieces of this solid frame-work, which gives to the body its form, which permits and limits its movements. Isolate, the better to know them, those levers, those springs, and those numerous powers it possesses, to transmit the orders of the will; unrol these tortuous tubes, filled with a regenerating fluid; pursue these sensible and delicate fibres, which establish such intimate relations between us and the bodies which surround us, with a rapidity swifter than lightning. Analyze those optical, chemical, and accoustic instruments, which produce all the sensations. Then behold these organs, animated under the influence of life; execute a series of functions, excelling each other in wonder, and connected with each other by an admirable, although invisible, chain.

Afterwards extend a scrutinizing eye on the beings around you, the study of which may enlighten that of ourselves; on those elements which are to be identified with our own bodies, and serve for their sustenance; and on those deleterious substances which tend to destroy it.

In fine, learn to know the best works, and how a medical man ought to compose a good library. At this period of your studies, you will attend in this amphitheatre, and hear the secrets of the science developed. Learn that, even in the intercourse with the Faculty, other means are still offered you for practical exercises in the laboratories, where places are assigned you, in order that, by your own researches, you may ascertain the facts advanced by your masters. Some of your old fellow pupils, selected from our practical schools, are specially charged with the care of directing your inexperience, to transmit and develope the principles they have derived from the same source. A museum of anatomy, a botanic garden, an admirably chosen medical library, a chemical laboratory, a cabinet of physics, a most complete collection of materia medica, and a surgical *arsenal*, are all open to you—for you they display all their treasures, and all the resources of which science makes use.

Under the protecting ægis of your instruction, you will be received in the clinical studies. There able professors will form you to the art and observation of the practice of medicine. Frequent quent assiduously those asylums of pain where the disorder of one alone serves for the instruction of all; where facts the most rare, and circumstances the most important, offer themselves to the annals of science, as in the celebrated temple of Greece, where details, suspended on the columns, related the history of past disorders in the hope of instructing for the future.

The prizes bestowed were for anatomy and physiology, internal and external pathology, anatomy, female pupils in midwifery, chemistry, &c.—1200 pupils were present.

---

*Periodical Menstrual Flux observed in Women of an advanced Age.*—On the cessation of the menses in some women, there is established a periodical flux, more or less sensible, of a different nature and form, which generally assume the menstrual type.

Margaret Flimaux, of a sanguine temperament, had her courses at 14, which regularly continued till 40 (excepting when pregnant, and giving suck). At this period they ceased, but she felt violent pains in the abdomen, which terminated by a vomiting of blood, returning periodically every month to the age of 70, without troubling the digestive or other functions. At this new epoch, the blood resumed its course by the organs of generation; and this periodical flux continued to take place every month, diminishing gradually to the age of 95, when she died of an adynomic fever. She preserved, during her long career, a remarkably fresh complexion, was extremely lively, lived frugally, and was passionately fond of milk.

The wife of the celebrated surgeon M. Cuisignier, offered an analogous phenomenon; and at present there is existing, in the commune of Bellenglize, a woman of 80, Margaret D'Elparte, who has every month a flux of several ounces of blood by the organs of generation.

We leave to those who love hypotheses, to explain these facts as they please, or as they can: we content ourselves with having observed and narrated them. SARRAZIN.

---

In consequence of the premature death of the late Mr. Royston, who had engaged to deliver the Anniversary Oration before the Medical Society of London, on the 8th of next March, Mr. Stevenson has complied with the earnest request of the President and Council, to become, on a very short notice, the representative of our late worthy coadjutor. As this undertaking, before such an audience, cannot be considered trifling, the Council have expressed their thanks in a manner becoming the occasion.

---

We have received from a publisher an account of Mr. J. BELL's health, and of his future intentions. If that gentleman will favour us with the history of his illness, we have no doubt, that, coming from an experienced physiologist, it will afford improvement to our readers and advantage to the public.

At the sittings of the Faculty of Medicine of Paris, of the 26th December last, there was exhibited a remarkable case of Polysarchia, or extreme corpulency, in a child three years and a half old. Those who have seen Daniel Lambert may form a tolerable idea of the child, who enjoys perfect health, and walks tolerably well; is lively; and displays a precocity of intellect. The features have already acquired a maturity of form; and, to see the face alone, one might suppose the subject upwards of 20. The corpulency is regular throughout the system; and the tone of the muscles firm. His parents are both thin, and of small stature;—the father 5 feet, the mother 4 feet 6 inches, and weighing only 70 pounds, whereas the child at present weighs upwards of 140 pounds, and is about to be exhibited publicly as a spectacle. On turning up his clothes, he would appear to want the external parts of generation; and it was only by laying him on his back, and extending his thighs, that the parts could be discovered. They are perfect, but extremely small, arising, no doubt, from the want of air, and the perpetual compression they undergo.

---

The observation made formerly by Thilenius, in very violent and long lasting tooth-ach, that the gums are surrounded by a very red edge, as broad as a straw, just over the painful place, in a manner as if it were a ribband, has been confirmed by Mr. Steinbruch. He has not only observed this symptom several times in violent tooth-ach, but also in several other painful affections of the head, particularly the face-ach of women; and found himself persuaded by it to subject such patients to the antiphlogistic treatment, notwithstanding their debility, and the irritability of their constitution. Bleeding and leeches he found, however, less serviceable than nitre, in larger or smaller proportions, which, if the digestion of the patients was much injured, or their stomach too sensible, he combined with mucilaginous remedies.

---

Dr. Harles distinguishes a particular sort of epilepsy, the primary cause of which is said to be an affection of the spinal marrow, from that were it depends on affections of the brain, or the epilepsia cerebralis. The symptoms characterising the former, says he, are, that the first paroxysm is felt more distinctly in the primarily affected part of the body, or in the gastric region, the sensorium not being so much, and more transitorily, affected; so that recollection is not entirely lost, but that the sensorium only afterwards bears a part in the disorder by consensus; and that all other symptoms shew less energy. The occasional causes of this species are, an accumulation of sanguineous or seroso-lymphatic matter in the region of the spinal marrow, or the intercostal and splanchnic nerves connected with it.

Mr.

Mr. Clarke will commence his next Course of Lectures on Midwifery, and the Diseases of Women and Children, on Thursday, March 20th. The Lectures are read every morning, from a quarter past ten to a quarter past eleven, for the convenience of students attending the Hospitals.

---

Dr. Burrows intends publishing, in a few days, Cursory Remarks on a Bill, now pending in Parliament, for Regulating Mad-Houses, throughout Great Britain; and upon its probable Influence on the physical and moral Condition of the Insane, and upon the Interests of those concerned in their care and management.

---

### REPORT OF DISEASES.

The mildness of the season, as was long ago remarked by Dr. Heberden, has proved particularly favourable to health. The diseases, however, still continue inflammatory, and require the same early and rapid depletion as heretofore. Hæmorrhage has been frequent, as is usual in the spring. A young female, in the higher ranks of life, has been destroyed by epistaxis. The disease was much exasperated by the loss of her mother, in consequence of which a despondency followed, which seemed to supersede all the restorative powers of the economy.

The inflammatory complaints have been chiefly enteritis and peritonitis. Pleurisy has been less frequent than usual in the spring. One case of enteritis arose from long constipation. The patient was bled till the surgeon became alarmed at two fits of convulsion, and an intermitting pulse. By this time the abdomen, which had been tender and tense, was become flaccid, and would bear pressure: the hiccough, which had been teizing for more than twenty hours, with only short intervals, ceased; and by degrees the pulse became steady, but very quick and small, as after an hæmorrhage. The sickness had ceased, so that cathartic pills remained on the stomach. Three diluting clysters had been thrown up without being returned.

In this state it was thought adviseable to trust to the remedies then in the intestinal canal, only placing a suppository in the rectum. A very small quantity of wine was permitted, if the patient should be in danger of fainting. This instantly renewed the vomiting, and was discontinued by the patient's desire. The bowels, however, now began to act freely; and, after the second evacuation, lumps of fæces were voided broken, and appeared slightly dissolved in the clysters. After this the patient recovered rapidly.

Catalogue

